# Estrogen level monitoring in artificial frozen-thawed embryo transfer cycles using step-up regime without pituitary suppression: is it necessary?

**DOI:** 10.1186/1743-1050-5-4

**Published:** 2008-07-04

**Authors:** Zhihong Niu, Yun Feng, Yijuan Sun, Aijun Zhang, Huiqin Zhang

**Affiliations:** 1 IVF-unit, Department of Obstetrics and Gynecology, RuiJin Hospital Affiliated to Shanghai Jiaotong University, PR China; 2 Shanghai Institute of Planned Parenthood Research, Fudan University, Shanghai, PR China

## Abstract

**Background:**

To discuss the meaning of serum oestradiol monitoring in frozen embryo transfer cycle using hormone replacement without pretreatment with gonadotropin hormone (GnRH) agonist.

**Methods:**

The data from two hundred twelve women undergoing two hundred seventy-four frozen-thawed embryo transfer (FET) cycles was included in this retrospective cohort study. They were detected of serum oestradiol levels and endometrium thicknesses during hormone supplement FET cycles and compared their pregnancy outcomes according to their oestradiol level on progesterone initiation day.

**Results:**

Patients with different levels of serum oestradiol (percentile 0–25^th^, 25^th^–75^th ^and 75^th^–100^th^) on progesterone initiation day yielded the endometrium thickness of 9.3 ± 0.12, 8.9 ± 0.07 and 9.1 ± 0.11 mm(P > 0.05) and the pregnancy rate of 32.2%, 38.4% and 36.3% (P > 0.05) respectively.

**Conclusion:**

The serum estradiol level did not predict pregnancy success in hormone replacement FET cycles, suggesting that oestradiol monitoring in this method of endometrial preparation is unnecessary.

## Background

In in vitro fertilization treatment, the transfer of frozen embryos has been a good option to improve accumulated pregnancy rate and avoid ovarian hyperstimulation syndrome (OHSS). Frozen embryo transfer in women with functioning ovaries can be timed with ovulation in natural cycle or after artificially preparing the endometrium with exogenous hormones. Although natural cycle protocol was often preferred by many patients for afraid of hormones' side effect, problems often occur when this protocol is used. The accurate monitoring of the cycle required to determine ovulation entails higher costs of money and time. The exact timing of ovulation is often difficult to determine, particularly in women with irregular cycle or poor follicle development, so the risk of cycle cancellation is therefore high.

Artificial endometrial preparation with exogenous steroids has some important advantages. Doctors and patients can select the timing of embryo thawing and transfer, and the possibility of cycle cancellation can be drastically reduced. Many published articles have proved the same as or higher pregnancy rate of hormone replacement cycle than natural cycle [1,2]. There are two sorts of artificial endometrial preparation: using GnRH agonist to obtain a previous ovarian suppression or not. The aim of pretreatment with GnRHa is to avoid spontaneous ovulation, but it is costly and there is a risk of hypoestrogenic side effect that would lengthen the preparation. Many published studies compared the two artificial protocols and showed the similar clinical outcome [2-5]. Therefore, clearly the program of absent of GnRH agonist was more preferred because of its simple and low cost.

It has been known that in stimulation cycle, concentration of serum E_2 _had important roles in regulation gonadotropin doses and prediction of outcomes. In hormone replacement FET cycle without pituitary, exogenous estrodiol would lead to a rise in serum E_2_, similar to that observed before ovulation. Whether the hormone's level have any meaning in deciding the timing of ET or predicting the possibility of pregnancy? To date, few articles have reported the question. The present retrospective study was tending to discuss the meaning of E_2 _concentration in frozen embryo transfer cycle using hormone replacement without GnRHa.

## Methods

### Patients

From January 2004 until July 2007, all the patients who underwent the transfer of their frozen-thawed embryos that were cryopreservated in previous IVF or ICSI cycle and preferred hormone replacement protocol were included in this retrospective study. The study group consisted of 136 women with functioning ovaries who underwent 176 cycles of frozen-thawed ET. 33 of the patients hadn't fresh embryo transfer for avoiding OHSS. There were 155 ovulatory and 57 anovulatory patients in the group and they all preferred artificial endometrial preparation for lower cancellation rate.

### Embryo freezing-thawing

On the 3^rd ^day after oocyte retrival, fresh cleavage-stage embryos generated using IVF or ICSI were assessed and assigned using a standardized scoring system: A) number of blastomeres (BL) was divided into four categories: 1 = four BL, 2 = five BL, 3 = six to seven BL and 4 = eight to ten BL. B) Degree of fragmentation (FR) was also scaled with a score of 4 indicating no fragmentation, 3 representing ≥ 10%, 2 means 11–25%, 1 indicating 26 – 50%, and 0 refers to > 50%; and C) Equality (EQ) or variation in the sizes of BL was categorized as 1, uniform or almost uniform BL size or 0, varying BL size. The total score for an embryo included the three aspects (BL, FR, and EQ). If the score for an embryo reached 5, it was considered "a viable embryo". Only those viable embryos were selected to be cryopreservated according to a protocol previously described using 1,2-propanediol and sucrose solution in phosphate-buffered saline as cryoprotectants[6]. Thawing was preformed by the transfer of cryotubes into a warm bath at a temperature of 35°C. After complete thawing, the embryos were processed through a series of decreasing concentrations of propanedion and sucrose, washed 3 times in phosphate-buffered saline, and placed in fresh equilibrated, warmed culture medium. Embryos surviving the freezing procedure (≥ 50% of their initial number of blastomeres intact) were then transferred with 1 hour. Intrauterine transfer of thawed embryos was scheduled after artificial endometrial preparation.

### Endometrial preparation and ET

All patients started estrogen valerianic (progynova^®^, Schering, German) treatment on the 2^nd ^day of their menstrual period without previous down regulation of gonadotropin. The standard E replacement was 2 mg pre day, 5 days and 3 mg per day, 5 days. 10 days later, patients' endometrial thickness was evaluated through vaginal ultrasound. If the thickness was ≥ 8 mm, the same dose of estrogen was continued for another 4 days and if not, the doses increased to 4 to 6 mg/d, given per os in 2 divided doses for another 10 days by step-up. On the day of thickness reaching 8 mm, progesterone was injected 40 mg/d, 2 days and changed to 40 mg, bid.

If the endometrium didn't reach the thickness of 8 mm after 20 days of continuous E administration, the cycle was canceled. Embryos were transferred after 72 h after the initiation of progesterone. The same doses of eatrogen and progesterone were continued until obtaining a serum β-hCG assay 11 days after ET. If the pregnancy test was positive, the hormone replacement went on another 8 weeks and patients were followed with serial ultrasonography to determine fetal viability. Clinical pregnancy was defined as the presence of a gestational sac on transverginal ultrasound.

### Hormone assay

Serum level of FSH (IU/L), LH (IU/L), E2 (pg/ml) and P (ng/ml) were measured in all patients on the day of E initiation (day 2), LH and E2 levels were measured on the days ultrasound was done, the day of P administration and the day of ET. Blood was drawn at 8 AM, 10 hours after the last doses of hormone. Hormone assay was performed by commercially available kits with use of Fluorescence Polariza immunassay (FIPA). by the Abbott AXSYM^® ^assay(Abbott Labs, Abbott Park, IL, USA). The inter- and intra-assay coefficients of variation were all <10%.

### Statistical Analysis

Statistical Package for Social Sciences (SPSS v 13.0 for windows, Chicago, IL) software was used for data analysis. Descriptive statistics were performed for each variable; quantitative results are presented as the mean (± SD). Means were compared by using one way ANOVA and two-sample *t*-test. Proportions for the two groups were compared by using the *χ*^2 ^test and the Fisher exact test. *P *< 0.05 was considered statistically significant.

## Result

Between January 2004 and October 2007, 212 patients underwent 274 artificially prepared frozen embryo transfer without prior pituitary desensitization using GnRHa and all embryos available for transfer originated from the same fresh cycle. Mean age of the patients in fresh cycle and frozen embryo transfer were 29.3 ± and 30.1 ± respectively, mean duration of embryos cryopreservation before being thawed and transferred were 7.3 ± 3.5 months. In frozen embryo transfer cycle, the mean days of exogenous E2 supplementation before progesterone administration were 18.3 ± 2.9 and the mean endometrium thickness on progesterone initial day was 8.5 ± 0.3 mm. The serum LH and E2 concentration on progesterone day was 13.5 ± 2.8 IU/ml and 197.2 ± 65.5 pg/ml respectively. The averange number of embryo transferred was 2.5 ± 0.3 and the total clinic pregnancy was 41.2%.

To discuss whether different levels of E2 would result in different outcomes, patients were classified according to serum E2 concentrations percentile (group 1: 0–25^th^, group 2: 25^th^–75^th ^and group 3: 75^th^–100^th^) on the day of progesterone injection. The frozen embryos had been originated from some stimulation cycle. Table 1 details the patients' characteristic of each group in stimulation cycles. There were no differences in age, basal FSH, total gonadotrophin doses, No. of oocytes retrieved, fertilization rate and No. of embryos frozen between groups. Table 2 listed the characteristic of groups in frozen-thawed embryo transfer cycle. All of the indicators were similar between groups, including duration of embryo cryopreservation and estrogen administration, embryo survival rate and No., score of embryo transferred and clinical outcomes. Table 3 compares the patients' E2 levels and endometrium thickness on day 2, day12 and the day of progesterone initiation. The hormone levels of the three groups show differences from day12 and although the varieties of E2 level, their endometrium had the same thickness.

**Table 1:** Patients and stimulation cycle characteristic categorized according to percentile analysis of E2 levels measured on P initiation day

	Groups of cycles according to E2 level percentile			*p*
		
	Group1 (n = 55)	Group 2 (n = 135)	Group 3 (n = 84)	
Age at stimulation(years)	30.7 ± 3.7	29.9 ± 3.9	30.1 ± 3.5	NS
Basal serum FSH level(IU/L)	5.9 ± 2.8	6.3 ± 2.5	5.8 ± 2.3	NS
Total Gn doses(IU)	26.3 ± 7.5	27.0 ± 8.3	26.5 ± 7.8	NS
No. of oocyte retrieved	18.1 ± 8.1	18.9 ± 8.6	17.3 ± 7.8	NS
No. of oocytes fertilized normally	13 ± 5.2	14.6 ± 5.8	13.2 ± 5.5	NS
No. of embryos frozen/cycle	5.7 ± 2.1	6.8 ± 2.6	6.5 ± 2.3	NS

**Table 2:** Patients' characteristic in FRET cycle categorized according to percentile analysis of E2 levels measured on P initiation day

	Groups of cycles according to E2 level percentile			*p*
		
	Group1 (n = 55)	Group 2 (n = 135)	Group 3 (n = 84)	
Duration between freezing and thawing(month)	8.0 ± 3.8	7.2 ± 3.0	7.7 ± 3.4	NS
Mean basal serum FSH level(IU/L)	5.2 ± 2.1	6.3 ± 2.4	5.8 ± 2.6	NS
Total estradiol doses(mg)^a^	44.8 ± 5.5	43.9 ± 5.8	44.2 ± 5.7	NS
Duration of estradiol supplementation	18.4 ± 2.3	18.8 ± 2.2	18.0 ± 2.1	NS
No. of embryos thawed/cycle	2.9 ± 0.5	3.3 ± 0.4	3.2 ± 0.5	NS
No. of embryos surviving/thaw^b^	2.5 ± 0.3	2.6 ± 0.2	2.4 ± 0.4	NS
Post-thaw embryo survival rate(%)	86	82	89	NS
Endometrial thickness(mm)^a^	8.6 ± 0.1	8.3 ± 0.2	8.7 ± 0.3	NS
Embryo score(mean)	7.1	7.3	7.2	NS
Pregnancy rate(%)	44	40.8	41.9	NS
Implantation rate(%)	26.2	23.1	22.5	NS
On going pregnancy rate(%)	32.2	38.4	36.3	NS

^a ^on the day of progesterone administration

^b ^all the survival embryos were transferred in to uterus

**Table 3:** E2 levels and endometrium thickness categorized according to percentile analysis of E2 levels measured on P initiation day of an artificial endometrium preparation cycle for FRET

day	E2 level(pg/ml)			p	Endometrium thickness(mm)			p
				
	Group 1	Group 2	Group 3		Group 1	Group 2	Group 3	
Day2	43 ± 12.4	52 ± 15.5	50 ± 17.9	NS	5.5 ± 1.9	5.8 ± 2.0	5.7 ± 1.9	NS
Day12	90.4 ± 18.4	121.8 ± 23.5	156.4 ± 39.6	0.03	6.9 ± 0.18	6.7 ± 0.11	7.0 ± 0.14	NS
Day of P initiation	110 ± 20.5	191.9 ± 27.7	299 ± 48.9	< 0.01	9.3 ± 0.12	8.9 ± 0.07	9.1 ± 0.11	NS

## Discussion

The success of a frozen-thawed embryo transfer program is closely linked to exact synchronization between endometrial maturation and embryo development [7]. Such synchronization may be achieved in a natural cycle after spontaneous ovulation [7,8] or after artificial preparation of the endometrium with exogenous steroids [8,9].

In artificial endometrium preparation cycle without suppression by GnRH agonist, it's very important to start estradio treatment in the early follicular phase (on day 1 or day 2) in order to inhibit spontaneous ovulation. Although high fixed doses of estradiol and step-up protocol had the likely effect, we choose the latter considering that more physiologic estrogenic stimulation of endometrium that mimics the hormonal pattern of spontaneous cycle is more suitable for endometrial development. In this type of endometrium preparation program, endometrium thickness is the key to be monitored. A receptive endometrium should reach a thickness of at least 5–8 mm [10]. Above this threshold, the endometrium can maintain its receptivity for up to 40–60 days, as has been shown by oocyte donation programs. Navot et al [11] reported that shorter (5–10 days) and lower dosage protocols of estradiol priming of the endometrium could result in higher abortion rates. Borini et al also found in a proliferative phase of less than 10 days are related to a higher abortion rate [12]. This indicates an optimal endometrial proliferation which is necessary to enable optimal development of progesterone receptors and subsequent transformation into an endometrium receptive to the transferred embryo. Devroey and Pados [13] presumed that an adequate period of estrogen administration is necessary in order to achieve a subsequent normal secretory endometrium. In our program, as a step-up protocol, we usually controlled the duration of proliferative phase in 15–21 days, even the thickness had reached 8 mm before that time.

The present retrospective study addressed the question whether different levels of E_2 _on progesterone initial day are associated with the likelihood of outcomes, in patients undergoing a FET cycle in which endometrium is prepared with hormone replacement in the absence of pituitary down regulation. The data presented herein show that when given enough duration of estradiol administration, different E_2 _levels on progesterone initial day yielded the similar pregnancy and implantation rate. On the other hand, the thickness of endometrium had not relationship with E_2 _concentration. The information might be useful in clinical practice since it indicates that a wide range of E2 levels is compatible with establishment of clinical pregnancy, and thus there is no value in measuring E2 levels during a FET cycle. Our findings also corroborate observations by other investigators. Remohi et al [10] suggested in 1997 that neither endometrial thickness nor serum estradiol were able to predict optimal receptivity and therefore outcome in oocyte donation. From the experience of Simon et al [1], the serum E_2 _level was an unimportant index when the decision for ET had to be made, and therefore only the measurement of endometrium thickness by ultrasound was need. Banz et al [14] found that endometrial thickness between 7 and 15 mm and estradiol serum level did not relate to significantly different pregnancy rates. So they concluded that monitoring is unnecessary in cryopreservation cycles with a protocol using estradiol patches and progesterone vaginal gel since the percentage of the patients with an endometrial thickness <7 mm or >15 mm was only 4.8%.

Remohi J et al [15] reported that in oocyte donation cycles, serum oestradiol on centrations < 100 pg/ml are able to induce changes sufficient to sustain normal implantation, and when those cycles with serum oestradiol, 100 pg/ml were further explored, they found normal implantation at concentrations,50 pg/ml, which in natural cycles is considered to be the limit for ovarian arrest.

The crucial role of steroid hormones in preparing and maintaining the endometrium for successful embryonic implantation is beyond any doubt. However, unlike progesterone, oestradiol requirements for implantation in humans are very low. The majority of authors appear to agree with the concept that oestradiol is permissive but not essential in mammals that do not undergo dispause [16-19]. Our clinical study suggestted the existence of a wide oestradiol window which allows the endometrium to prepare itself for implantation. According to Simon et al [20], the oestradiol window reflected a permissive hormonal effect rather than a direct action performed at the paracrine/autocrine concentration by cytokines and adhesion molecules. This may be the reason for the absence of a relationship between oestradiol concentrations and implantation at the clinical concentration. The serum estradiol level did not predict pregnancy success in hormone replacement FET cycles, suggesting that oestradiol monitoring in this method of endometrial preparation is unnecessary."

In conclusion, our study suggest that in E_2 _step-up artificial endometrium preparation protocol without pituitary down-regulation, assessment of E_2 _level on progesterone initial day does not appear to yield useful information in predicting pregnancy success. So, it is therefore possible that our protocol can be further simplified by performing only ultrasound tests and a single measurement of serum P on the day of P initiation.

## Competing interests

The authors declare that they have no competing interests.
